# Evaluating the Effectiveness of France’s Indoor Smoke-Free Law 1 Year and 5 Years after Implementation: Findings from the ITC France Survey

**DOI:** 10.1371/journal.pone.0066692

**Published:** 2013-06-21

**Authors:** Geoffrey T. Fong, Lorraine V. Craig, Romain Guignard, Gera E. Nagelhout, Megan K. Tait, Pete Driezen, Ryan David Kennedy, Christian Boudreau, Jean-Louis Wilquin, Antoine Deutsch, François Beck

**Affiliations:** 1 Department of Psychology, University of Waterloo, Waterloo, Ontario, Canada; 2 School of Public Health and Health Systems, University of Waterloo, Waterloo, Ontario, Canada; 3 Ontario Institute for Cancer Research, Toronto, Ontario, Canada; 4 French Institute for Health Promotion and Health Education (INPES), Saint-Denis, France; 5 Maastricht University (CAPHRI), Maastricht, The Netherlands; 6 STIVORO Dutch Expert Centre on Tobacco Control, The Hague, The Netherlands; 7 Propel Centre for Population Health Impact, University of Waterloo, Waterloo, Ontario, Canada; 8 Department of Health, Behavior and Society, Johns Hopkins Bloomberg School of Public Health, Baltimore, Maryland, United States of America; 9 Department of Statistics and Actuarial Science, University of Waterloo, Waterloo, Ontario, Canada; 10 French Institute for Health Promotion and Health Education (INPES), Saint-Denis, France; 11 French National Cancer Institute (INCa), Paris, France; 12 Cermes3-Cesames team (Research Centre Medicine, Sciences, Health, Mental Health, Health Policy), University of Paris Descartes, EHESS, Paris, France; The University of Auckland, New Zealand

## Abstract

France implemented a comprehensive smoke-free law in two phases: Phase 1 (February 2007) banned smoking in workplaces, shopping centres, airports, train stations, hospitals, and schools; Phase 2 (January 2008) banned smoking in hospitality venues (bars, restaurants, hotels, casinos, nightclubs). This paper evaluates France’s smoke-free law based on the International Tobacco Control Policy Evaluation Project in France (the ITC France Project), which conducted a cohort survey of approximately 1,500 smokers and 500 non-smokers before the implementation of the laws (Wave 1) and two waves after the implementation (Waves 2 and 3). Results show that the smoke-free law led to a very significant and near-total elimination of observed smoking in key venues such as bars (from 94–97% to 4%) and restaurants (from 60–71% to 2–3%) at Wave 2, which was sustained four years later (6–8% in bars; 1–2% in restaurants). The reduction in self-reported smoking by smoking respondents was nearly identical to the effects shown in observed smoking. Observed smoking in workplaces declined significantly after the law (from 41–48% to 18–20%), which continued to decline at Wave 3 (to 14–15%). Support for the smoke-free laws increased significantly after their implementation and continued to increase at Wave 3 (p<.001 among smokers for bars and restaurants; p<.001 among smokers and p = .003 for non-smokers for workplaces). The findings demonstrate that smoke-free policies that are implemented in ways consistent with the Guidelines for Article 8 of the WHO Framework Convention on Tobacco Control (WHO FCTC) lead to substantial and sustained reductions in indoor smoking while also leading to high levels of support by the public. Moreover, contrary to arguments by opponents of smoke-free laws, smoking in the home did not increase after the law was implemented and prevalence of smoke-free homes among smokers increased from 23.2% before the law to 37.2% 5 years after the law.

## Introduction

The public health harms associated with exposure to second-hand smoke have been well documented including premature death, lung cancer, heart disease, and coronary obstructive pulmonary disease (COPD) [1]–[4]. Comprehensive smoke-free laws, if well-designed and implemented with strong enforcement measures, have been shown to greatly reduce or eliminate second-hand smoke in public venues [5]–[8]. Article 8 of the WHO Framework Convention on Tobacco Control (FCTC), which France ratified in 2004, obligates Parties to adopt effective measures to provide protection from exposure to second-hand smoke. Guidelines for Article 8 recognize the importance of monitoring and evaluation of smoke-free policies including assessing support for smoke-free policies and enforcement of and compliance with smoke-free policies [9].

Smoke-free policies were implemented in two phases in France. Phase 1 was implemented in February 2007 in workplaces, shopping centres, airports, train stations, hospitals, and schools. In January 2008, the ban was extended in Phase 2 to hospitality venues (cafés, bars, restaurants, hotels, casinos, and nightclubs). Media campaigns were held in France prior to the launch of each phase of the ban to inform the public about the forthcoming policies and to provide education about the health effects associated with exposure to second-hand smoke.

The International Tobacco Control Policy Evaluation Project in France (the ITC France Project) was created in 2006 to evaluate France’s smoke-free legislation as well as other tobacco control policies implemented under the FCTC including health warning labels, price and taxation policies, tobacco advertising and promotion bans, cessation strategies, and education campaigns [10]. The ITC France Project is part of the International Tobacco Control Policy Evaluation Project (the ITC Project) – an international research collaboration across more than 20 countries whose primary objective is to evaluate the psychosocial and behavioural effects of FCTC policies. The ITC Project is conducting large-scale prospective cohort surveys of tobacco users and non-users in countries inhabited by more than half of the world’s smokers [11]–[13]. For description of the conceptual model and objectives of the ITC Project, see Fong et al. (2006) [14]; for description of the survey methods, see Thompson et al. (2006) [15].

Three waves of a cohort survey of a nationally representative sample of smokers and non-smokers living in continental France have been completed between 2006 and 2012. The ITC France Wave 1 Survey was conducted just before the Phase 1 smoking ban was implemented in workplaces and other public venues in February 2007. The cohort was recontacted for the Wave 2 Survey after Phase 2 of the smoking ban. The Wave 3 Survey was conducted approximately 5 years after the ban in hospitality venues and 6 years after the workplace ban to provide an evaluation of the longer term impacts of the smoking ban and other policies.

The effectiveness of the smoking ban in France has been reported in the literature based on ITC France Wave 1 (pre-ban) and Wave 2 (post-ban) data [16]–[18]. However, the long-term effectiveness of smoke-free legislation in France has not yet been reported. Moreover, the post-ban effects from the perspective of non-smokers have not been reported before, as other studies have compared France with other countries that did not include a sample of non-smokers in their ITC Survey [16]–[17]. Long-term evaluations of smoke-free legislation in other European countries have largely focused on measuring second-hand smoke levels [19]–[20]. Cross-sectional population-based surveys have assessed the short- and medium-term effects of smoke-free legislation and support for future smoke-free policies [21]–[23].

This paper reports the results of the ITC France Survey evaluation of the 2007–2008 smoking ban across a variety of indoor public venues at one pre-implementation and two post-implementation time points. We report the impact of the ban on observed smoking and self-reported smoking behaviour in workplaces, restaurants, and bars and pubs; public support for comprehensive smoking bans in several public places; and the prevalence of smoking restrictions at home.

## Methods

### Ethics Statement

Prospective respondents were contacted via telephone (see below) and information was provided by the interviewer using a script built into the computer assisted telephone interview (CATI) program regarding the topic of the survey, the research institutions involved; prospective respondents were informed that all responses would be confidential. Respondents gave their consent verbally and the CATI script required the interviewer to enter the consent in the computer program before proceeding to the survey. As typical of telephone surveys, written consent was not obtained. However, prior to beginning the survey, the respondent was informed that he/she was free to decline to answer any question, and to terminate the survey at any time. The respondent was asked whether he/she could complete the survey at this time or at another more convenient time. All respondents were mailed compensation of a 10€ cheque (smokers for the 50-minute interview) or 8€ (non-smokers for the 30-minute interview) by mail after the survey interview (this was increased to 20€ at Wave 2 and Wave 3). Because of the cohort design, at Wave 2 and Wave 3, we recontacted all respondents from the previous wave by mail to inform them that they would be contacted by phone in the near future to invite them to participate in the next wave of the survey. When reached by phone, these recontact respondents were again read the information and consent script, and verbal consent was again obtained. The survey and research protocol was reviewed and cleared for ethics at each of the three survey waves by the Human Research Ethics Committee at the University of Waterloo, which waived the need for written informed consent from the participants.

### Respondents and Design

The ITC France Survey is a national longitudinal survey of adult (≥18 years of age) smokers and non-smokers. Smokers are those who have smoked more than 100 cigarettes in their life and smoke at least monthly. The CATI survey follows a random digit dialing (RDD) sampling design and covers continental France. To compensate for respondents lost to attrition, new respondents were randomly selected at Waves 2 and 3 using the same RDD sampling design and interview protocol as in Wave 1.

The questions used in the ITC France Survey were all adapted from the conceptual model and questionnaire of the ITC Four Country Survey.^14^ The process for translation of the English survey into French was guided by the translation protocol described by International Agency for Research on Cancer (IARC) which calls for discussion of translation among bilingual persons knowledgeable about the survey content.^12^ The ITC France team researchers in Paris met this qualification.

Wave 1 of the ITC France Survey was conducted by the survey firm Atoo between December 2006 and February 2007 with a cooperation rate of 75.3%. Wave 2 was conducted from September to November 2008 by the Institut de sondage Lavialle (ISL). Wave 3 was conducted from September to December 2012 by the firm BVA. The complete survey methodology can be found on the ITC Project website [24].

### Variables

Observed smoking in bars and restaurants was measured among those who had visited in the last 6 months with the questions “The last time you visited, were people smoking inside the café, bar or pub/restaurant?”. The response options were “yes/no”. Smoking behaviour in bars and restaurants was assessed with the question “Did you smoke at all at the bar/restaurant during your last visit, either indoors or outdoors?” Respondents who answered “yes” were asked whether they smoked inside the bar/restaurant, outside, or both.

Observed smoking in workplaces was measured among those who were employed outside the home in indoor workplaces with the question “In the last 30 days, have people smoked in indoor areas where you work?”. The response options were “yes/no”. Smoking behaviour in workplaces was measured with the question “In the last 30 days, have you smoked in indoor areas at work?” (yes/no).

Support for smoke-free law in bars and restaurants was assessed with the question “Do you support or oppose the French total ban on smoking inside cafés, bars and pubs/restaurants?” with options “strongly support/support/oppose/strongly oppose”. Support for a smoke-free law in cars with children was assessed with the question “Do you support or oppose a French total ban on smoking in cars with children with them?” with the same response options. Support for bans in other places was assessed with the question “For each of the following public places, please tell me if you think smoking should be allowed in all indoor areas, only in some indoor areas, or not allowed indoors at all.” Overall support for the smoking ban was measured with “Overall, would you say that the ban on smoking in restaurants, cafés and bars, and other public places is a good thing or a bad thing?” (very bad/bad/good/very good). Support for a ban in outdoor eating areas of restaurants was measured with the question “Do you think that smoking should be allowed in all outdoor eating areas, in designated outdoor eating areas such as smoking terraces, or not allowed in outdoor eating areas at all?” (yes/no). Support for a ban on smoking in cars with children was measured with “Do you support or oppose a French total ban on smoking in cars with children in them?”(strongly support/support/oppose/strongly oppose). Home smoking bans were measured with “Which of the following best describes smoking inside your home?” (anywhere inside/some rooms/never allowed anywhere/not allowed except under special circumstances).

### Analyses

Sampling weights were rescaled to population totals based on the prevalence of smoking in France. These inflated weights were then rescaled to the total number of smokers and non-smokers in the sample. Logistic regression models for longitudinal data (generalized estimating equations or GEE) were used to analyze differences in the measure of interest across waves. An unstructured within-group correlation matrix was used in the modeling to account for within-subject correlation. All of the models simultaneously controlled for gender, age at recruitment (categorized as 18–24, 25–39, 40–55, and 55+), wave, and smoking status (smoker vs. non-smoker). It has been documented that respondents who are newly recruited may vary in their responses compared to those who have completed one prior wave, who may vary from those who have completed two prior waves, and so on [25]–[27]. This variation in responses has been found in the ITC Surveys as well [28]. Therefore, time-in-sample, the number of times a respondent has participated in the survey, was also included in all of the models as a time-varying quantity over time to adjust for time-in-sample effects. An interaction term between wave and smoking status was also included in order to obtain separate model-based estimates for smokers and non-smokers adjusted for the covariates listed above. All p-values provided are adjusted for multiple comparisons using the Benjamini and Hochberg (1995) [29] method. Models were initially conducted including former smokers as an additional smoking status group; it was found that the responses of former smokers did not significantly differ from those of smokers. Thus, for all analyses presented here, former smokers were grouped with smokers. All analyses were run in SAS 9.2.

## Results

A total of 2260 (1735 smokers and 525 non-smokers) respondents completed Wave 1 of the ITC France Survey. A total of 2219 (1540 smokers, 164 former smokers, and 515 non-smokers) respondents completed Wave 2. Of these 2219 respondents, 1231 were from the cohort smoker sample and 414 were from the cohort non-smoker sample, resulting in an overall retention rate of 72.8%. The Wave 2 replenishment sample consisted of 473 smokers and 101 non-smokers. The cooperation rate among the Wave 2 replenishment sample was 80.5%. At Wave 3, a total of 2204 (1420 smokers, 297 former smokers, and 487 non-smokers) completed the ITC France Survey. Of these 2204 respondents, 1198 were from the cohort smoker sample and 390 were from the cohort non-smoker sample, which included 17 respondents who were non-smokers at Wave 2 and became smokers at Wave 3. There were 502 smokers and 114 non-smokers in the Wave 3 replenishment sample. The cooperation rate among the Wave 3 replenishment sample was 80.7%.The overall retention rate at Wave 3 was 71.6%. Note that 95% confidence intervals are presented in parentheses throughout the results.

### Observed Smoking and Smoking Behaviour in Key Venues

#### Restaurants

Among smokers, observed smoking in restaurants declined from 70.8% (63.1–77.5) before the law (Wave 1) to 2.0% (1.2–3.4) at first post-ban survey (Wave 2)reported by smokers and reported by non-smokers), 8 months after the law was implemented (p<.001). Observed smoking in restaurants among non-smokers also declined from 60.4% (50.8–69.3) at Wave 1 to 3.0% (1.4–6.2) at Wave 2 (p<.001). This very low prevalence of restaurants with smoking was sustained at the second post-ban survey (Wave 3) to 0.7% (0.4–1.4) among smokers and 2.1% (0.9–4.8) among non-smokers); this decline among smokers (from 2.0% (1.2–3.4) to 0.7% (0.4–1.4)) was statistically significant (p = .040). The slight decline from 3.0% (1.4–6.2) to 2.1% (0.9–4.8) among non-smokers was not statistically significant (p = .550).

**Figure 1:**
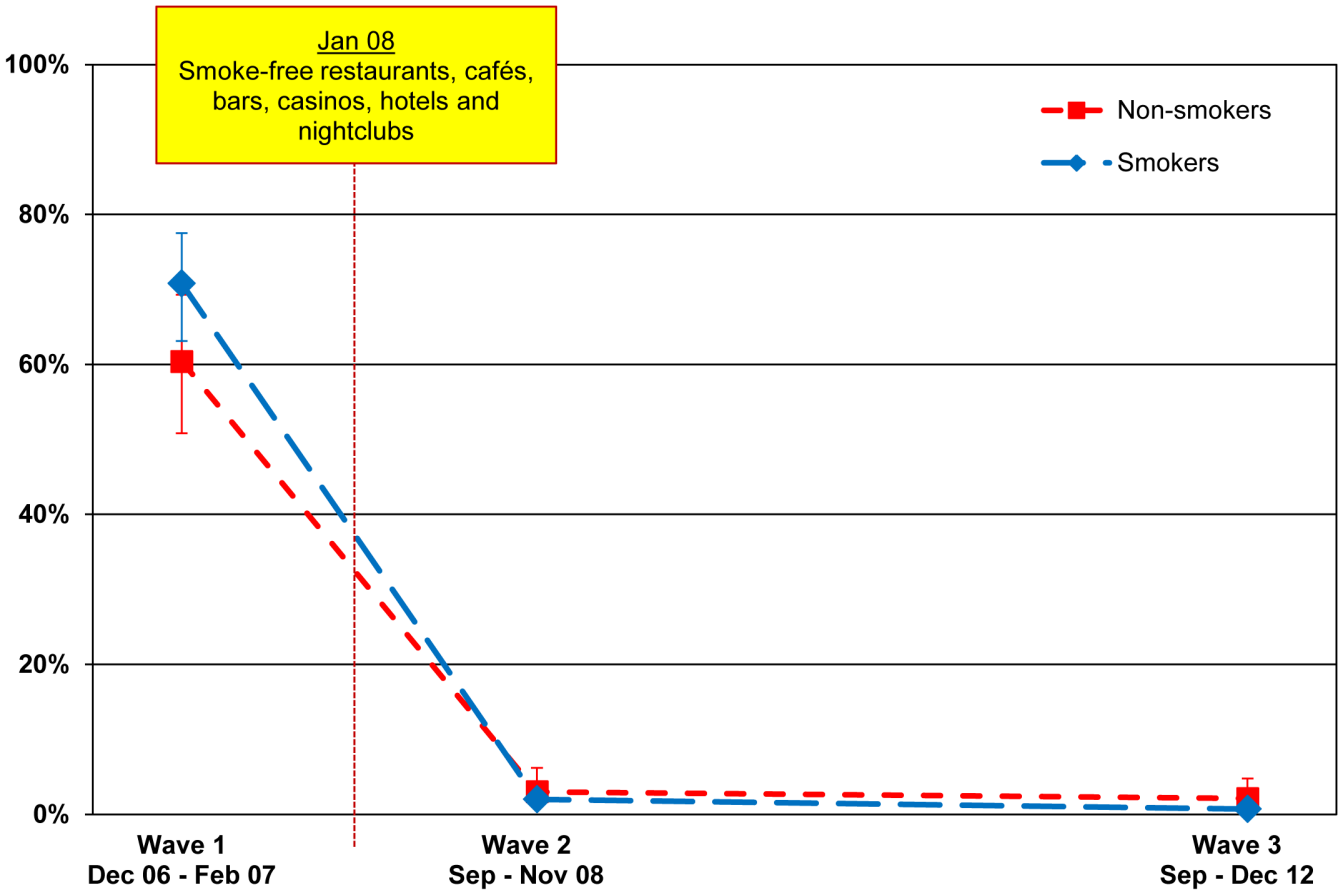
Noticing smoking inside restaurants. Percentage of smokers and non-smokers who noticed smoking inside restaurants among those who have visited these venues in the last 6 months, by wave.

These findings are presented in Figure 1.

We also examined self-reported smoking behaviour in restaurants. At Wave 1, 76.0% (67.4–82.9) of smokers smoked indoors or indoors and outdoors in a restaurant at last visit. This percentage decreased to 1.4% (0.7–3.0) at Wave 2 (p<.001) and was sustained at Wave 3 to 1.8% (1.0–3.4) (p = .622).

#### Bars

Observed smoking in bars declined from 96.6% (95.1–97.7) at Wave 1 to only 3.5% (2.4–5.0) at Wave 2 among smokers (p<.001). Among non-smokers, observed smoking in bars also declined from 93.5% (90.0–95.8) at Wave 1 to 4.4% (2.5–7.6) at Wave 2 (p<.001). The prevalence of observed smoking in bars at Wave 3 slightly increased to 6.4% (4.9–8.4) for smokers and 7.9% (4.8–12.7) for non-smokers. This slight increase was significant among smokers (p = 0.024), but not significant among non-smokers (p = .161).

**Figure 2:**
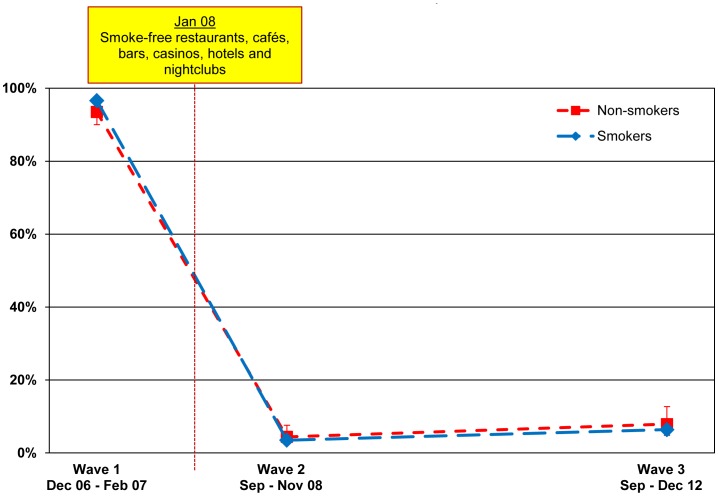
Noticing smoking inside bars, pubs, and cafés. Percentage of smokers and non-smokers who noticed smoking inside bars, pubs, and cafés among those who have visited these venues in the last 6 months, by wave.

These findings are presented in Figure 2.

Our analysis of self-reported smoking in bars found that at Wave 1, 95.5% (93.4–96.9) of smokers smoked inside or inside and outside a bar at last visit. This percentage decreased to 1.8% (1.0–3.2) at Wave 2 (p<.001). At Wave 3, 6.4% (4.5–9.2) of smokers reported smoking inside or outside a bar at last visit. This was significantly different from Wave 2 (p<.001) and Wave 1 (p<.001).

#### Workplaces

**Figure 3:**
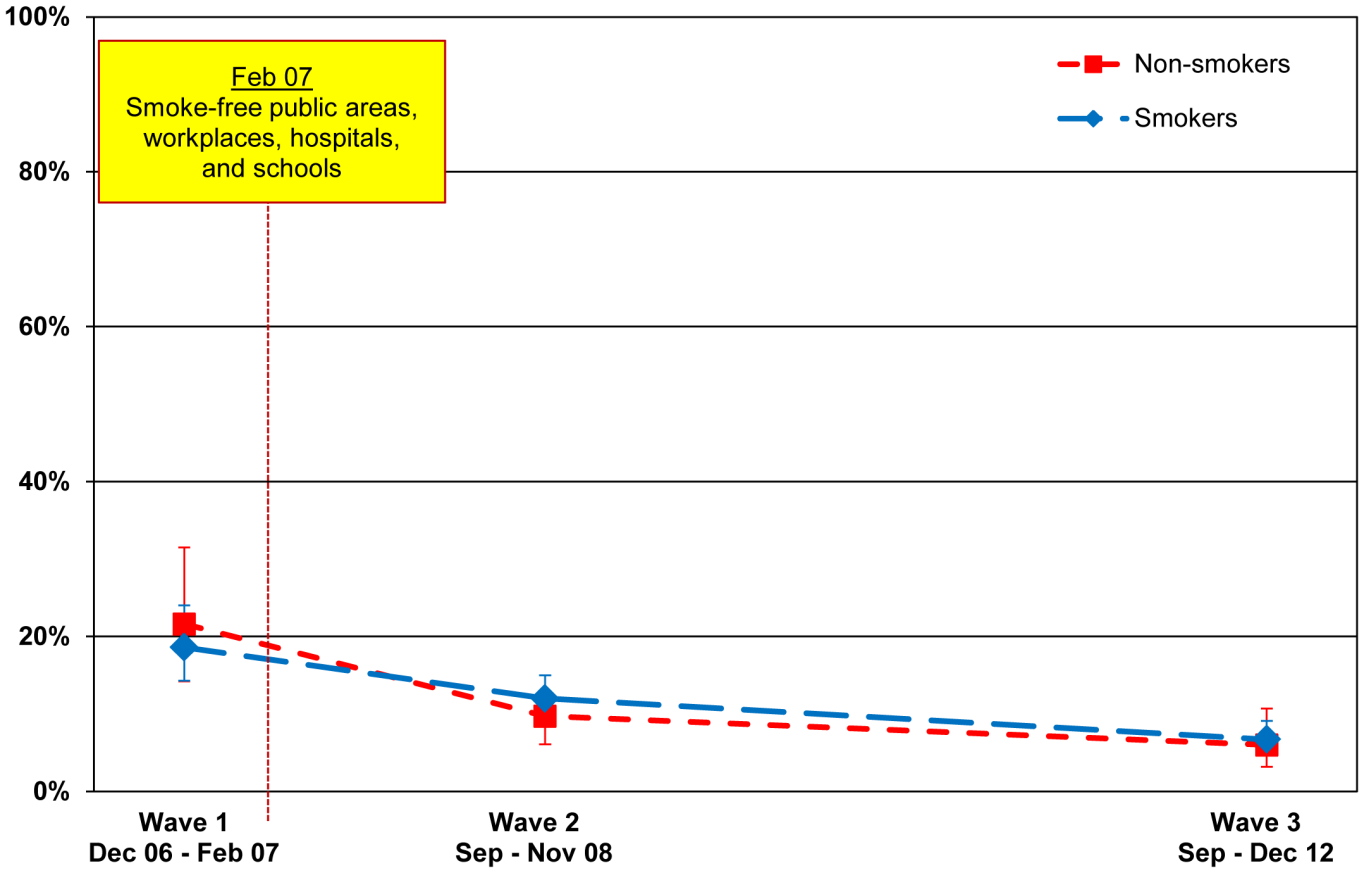
Noticing smoking at the workplace. Percentage of smokers and non-smokers who noticed smoking at the workplace in the last 30 days among those who reported that smoking is not allowed in any indoor area of their workplace, by wave.

Observed smoking in workplaces also declined significantly from 47.8% (42.9–52.8) for smokers and 41.1% (33.9–48.7) for non-smokers at Wave 1 to 20.0% (16.9–23.6) and 17.9% (13.2–23.9), respectively, at Wave 2 (p<.001 for both smokers and non-smokers). There was a further decline at Wave 3 to 14.5% (11.7–17.7) among smokers and 13.5% (9.1–19.6) among non-smokers, however the slight decrease was not significant for smokers (p = .056) and non-smokers (p = .340). The sample used in the analysis of observed smoking in the workplace included respondents who reported that smoking is never allowed in any indoor area, smoking is allowed in some inside areas (i.e., designated smoking rooms), and smoking is allowed in any indoor area. The presence of designated smoking rooms is a possible confounder, thus we also examined observed smoking in workplaces among those respondents who reported that smoking was not allowed in any indoor area of their workplace. For these workplaces, observed smoking also declined significantly from 18.6% (14.3–24.0) for smokers and 21.6% (14.2–31.5) for non-smokers at Wave 1 to 12.0% (9.5–15.0) and 9.7% (6.1–15.0), respectively, at Wave 2 (p = .023 for smokers; p = .019 for non-smokers). The slight decline at Wave 3 to 6.7% (4.9–9.1) among smokers was significant (p = .019), however the decline to 6.0% (3.2–10.7) among non-smokers was not significant (p = .352). These findings are presented in Figure 3. It should be noted that although the prevalence of smoking in workplaces is higher than the prevalence of smoking in public venues (e.g., restaurants, bars), the question for workplaces asked whether there was any smoking in the past 30 days, whereas the question for restaurants, bars, and other public places asked whether there was any smoking at last visit. It would be expected that the prevalences would be higher for workplaces all things equal because of the longer catchment period.

The analysis of self-reported smoking indoors in workplaces showed a similar trend. At Wave 1 39.6% (34.5–45.0) of smokers who worked outside the home in indoor workplaces smoked indoors regardless of their workplace smoking policy. This decreased to 14.1% (11.2–17.6) at Wave 2 (p<.001) and 11.8% (9.1–15.2) (p = .339) at Wave 3. Among those who reported a complete indoor workplace smoking ban at each wave, indoor smoking prevalence was low at Wave 1 (7.0% (4.5–10.8)) and decreased further at Wave 2 (4.6% (3.1–6.9)) and Wave 3 (3.3% (2.0–5.4)). However, the reductions at Wave 3 were not significant compared to Wave 1 (p = .104).

### Support for the Smoke-free Law in Key Venues

#### Restaurants

**Figure 4:**
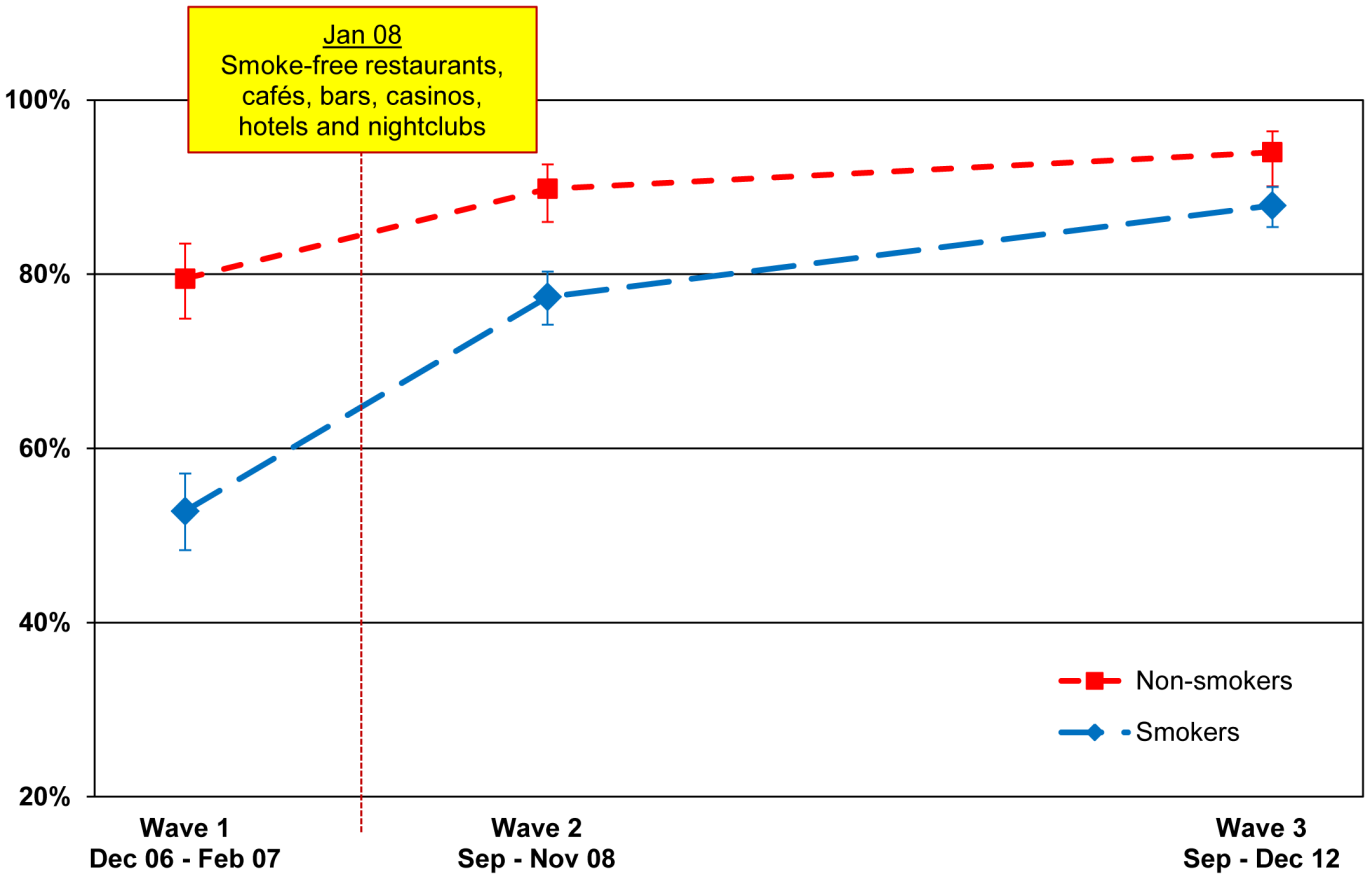
Support for smoking bans in restaurants. Percentage of smokers and non-smokers who support or strongly support smoking bans in restaurants, by wave.

Support for the smoke-free law in restaurants increased significantly after the law was implemented from 79.5% (74.9–83.5) to 89.8% (86.0–92.6) for non-smokers (p<.001) and from 52.8% (48.3–57.1) to 77.4% (74.2–80.3) for smokers (p<.001), with support continuing to increase at second post-ban follow-up for smokers (87.9% (85.4–90.0); p<.001) and for non-smokers (94.0% (90.1–96.4); p = .044). The pattern of the increase in support for smokers and non-smokers is presented in Figure 4. Of particular note is that whereas smokers were much less supportive of smoke-free restaurants before the law (a difference of 26.7 points), by the second follow-up, the gap was narrowed substantially (a difference of only 6.1 points), however the relative difference was not significant (p = .113).

#### Bars

**Figure 5:**
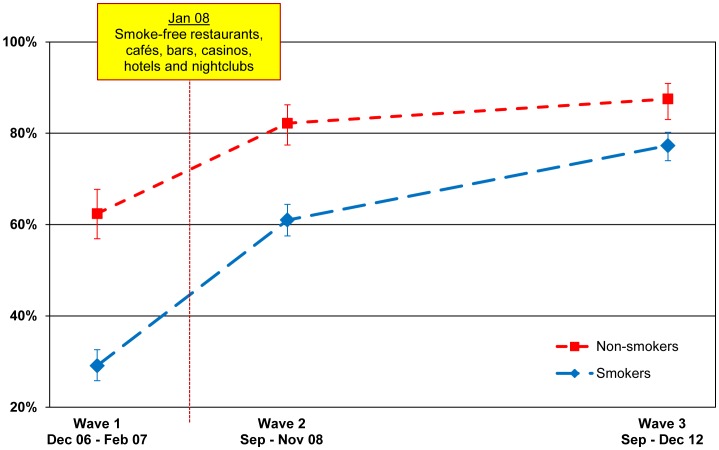
Support for smoking bans in bars, pubs, and cafés. Percentage of smokers and non-smokers who support or strongly support smoking bans in bars, pubs, and cafés, by wave.

As shown in Figure 5, support for smoke-free bars over time for smokers and non-smokers followed the same pattern as for restaurants, with support increasing significantly after the law was implemented (for smokers: 29.1% (25.8–32.6) to 61.0% (57.5–64.4), p<.001; for non-smokers: 62.4% (56.9–67.7) to 82.2% (77.4–86.2), p<.001), with support continuing to increase at second post-ban follow-up for smokers (77.3% (74.0–80.2) at Wave 3, p<.001) and non-smokers (87.5% (83.0–90.9) at Wave 3, p = .047). Again, whereas smokers were much less likely than non-smokers to support smoke-free bars before the law came into effect (a gap of 33.3%), the gap closed considerably at the second post-ban follow-up (10.2%). The relative difference between smokers and non-smokers from Wave 1 to Wave 3 is significant (p = .001).

#### Workplaces

**Figure 6:**
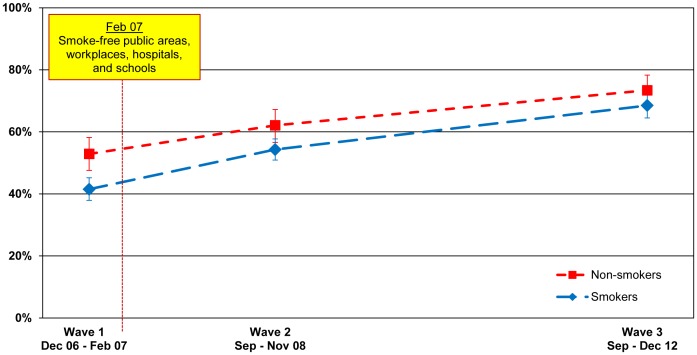
Support for smoking bans in the workplace. Percentage of smokers and non-smokers who reported that smoking should not be allowed indoors at all at the workplace, by wave.

Figure 6 shows that support for smoke-free workplaces increased significantly from 41.5% (37.9–45.2) before the smoke-free law was implemented (Wave 1) to 54.3% (50.9–57.7) among smokers after the law was implemented (p<.001). Among non-smokers, support for smoke-free workplaces also significantly increased from 52.9% (47.6–58.2) at Wave 1 to 62.1% (56.6–67.2) at Wave 2 (p = .019). Support continued to increase significantly at second post-ban follow-up to 68.5% (64.5–72.2) for smokers (p<.001) and 73.4% (67.8–78.3) for non-smokers (p = .003).

#### Other places

Table 1 shows that support for smoke-free public buses was extremely high (nearly 100%) at Waves 1 and 2 for both smokers and non-smokers. Support for smoke-free hospitals slightly increased between Waves 1 and 2 (86.9% (84.0–89.3) to 91.2% (89.2–92.8) for smokers, p = .043; 91.6% (88.0–94.3) to 94.8% (92.2–96.6) for non-smokers, p = .085) and remained at a very high level at Wave 3 (92.4% (90.3–94.1) among smokers and 95.2% (92.7–97.0) among non-smokers, non-significant). Support for smoke-free train stations significantly increased between Wave 2 (48.2% (44.8–51.6) for smokers; 48.7% (43.1–54.4) for non-smokers) and Wave 3 (60.4 (56.7–64.0) for smokers, p<.001; 60.3% (54.5–65.8) for non-smokers, p = .009). More than 75% of smokers and non-smokers reported that they supported the smoking ban in shopping malls and on trains at Waves 1 and 2. Support for smoke-free football stands was stable at slightly higher than 50% across the three waves among both smokers and non-smokers.

**Table 1:** Support for smoke-free laws in other venues, by smoking status (weighted population estimates).

	Smokers						Non-smokers					
Measure	N	W1 (%)	N	W2 (%)	N	W3 (%)	N	W1 (%)	N	W2 (%)	N	W3 (%)
Shopping malls†	1733	82.0 (78.9, 84.7)	1703	88.4 (86.0, 90.4)		Not asked	524	82.0 (77.3, 85.9)	515	87.8 (83.9, 90.9)		Not asked
Hospitals†	1734	86.9 (84.0, 89.3)	1704	91.2 (89.2, 92.8)	1717	92.4 (90.3, 94.1)	525	91.6 (88.0, 94.3)	515	94.8 (92.2, 96.6)	487	95.2 (92.7, 97.0)
On public buses†	1735	99.5 (98.9, 99.8)	1703	98.9 (98.0, 99.4)		Not asked	524	99.4 (98.5, 99.8)	515	99.2 (97.4, 99.8)		Not asked
On trains†	1735	76.5 (73.1, 79.6)	1703	78.7 (75.7, 81.4)		Not asked	525	83.6 (79.1, 87.2)	514	81.9 (76.5, 86.3)		Not asked
Train stations†	1733	42.1 (38.6, 45.7)	1704	48.2 (44.8, 51.6)	1715	60.4 (56.7, 64.0)	525	48.8 (43.4, 54.2)	514	48.7 (43.1, 54.4)	487	60.3 (54.5, 65.8)
Football stands†	1701	56.0 (52.4, 59.5)	1698	50.6 (47.1, 54.1)	1697	52.5 (48.7, 56.4)	516	52.8 (47.2, 58.3)	512	54.9 (49.4, 60.3)	482	55.4 (49.2, 61.5)
Outdoor eatingareas†		Not asked	1704	36.6 (33.3, 40.2)	1715	35.4 (31.4, 39.7)		Not asked	515	32.2 (27.1, 37.7)	487	35.2 (29.5, 41.5)
Cars with children*		Not asked	1703	88.6 (86.3, 90.5)	1709	89.2 (86.8, 91.1)		Not asked	514	90.4 (87.1, 92.9)	484	92.2 (89.2, 94.5)

Notes: The 95% confidence intervals are within parentheses. The total sample size (N) figures are unweighted; estimated percentages and 95% confidence intervals are weighted.

* Estimates are percent who support/strongly support the smoking ban.

† Estimates are percent who reported that smoking should not be allowed indoors at all.

### Overall Assessment of the Smoking Ban

At Wave 1, about 58.6% (54.1–62.9) of smokers said that the ban on smoking in public places was “a good thing” or “a very good thing” compared to 83.7% (79.2–87.4) of non-smokers (p<.001). At the first post-ban wave, these percentages were significantly higher for smokers (87.7% (85.6–89.5), p<.001) and for non-smokers (95.7% (93.6–97.2), p<.001). Support stayed high at the second post-ban follow-up for smokers (89.4% (87.2–91.3)) and non-smokers (96.9% (94.5–98.2)).

### Support for Future Possible Smoke-free Initiatives

The ITC France Survey at Wave 2 and Wave 3 included measures assessing support for possible future smoke-free initiatives in two areas: outdoor eating areas and smoking in cars with children (see Table 1).

#### Support for ban in outdoor eating areas

Support for a ban on smoking in outdoor eating areas remained stable between Wave 2 (36.6% (33.3–40.2) for smokers; 32.2% (27.1–37.7) for non-smokers) and Wave 3 (35.4% (31.4–39.7) for smokers; 35.2% (29.5–41.5) for non-smokers).

#### Support for ban on smoking in cars

The overwhelming majority of smokers and non-smokers at Wave 3 would support a smoking ban in cars with children (89.2% (86.8–91.1) vs. 92.2% (89.2–94.5), p = .385). There has been no change over time in this high level of support.

### The Impact of the Smoking Ban on Smoking Behaviour in Homes

**Figure 7:**
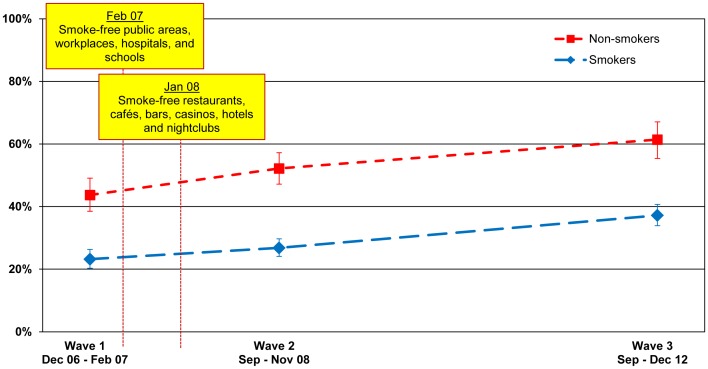
Smoking bans in the home. Percentage of smokers and non-smokers who reported that smoking is never allowed anywhere inside their home, by wave.

An argument sometimes made against smoke-free laws is that such laws would displace smoking from public places like bars/pubs into the home. If this were true, then this would represent an adverse consequence of such laws. We tested this possibility over the three waves of the ITC France Survey. As shown in Figure 7, bans on smoking in the homes of non-smokers increased from 43.7% (38.5–49.1) before the law (Wave 1) to 52.2% (47.2–57.2) after the law was implemented (Wave 2) (p = .006). Home smoking bans in non-smokers further increased at the second post-ban survey (Wave 3) to 61.4% (55.4–67.1) (p = .006). Among smokers, home smoking bans was stable between Wave 1 (23.2% (20.3–26.3)) and Wave 2 (26.8% (24.1–29.7)), but increased to 37.2% (33.9–40.7) at Wave 3 (p<.001).

## Discussion

The findings reported from three waves of the ITC France Survey over six years demonstrate clearly that the 2007–2008 smoke-free law led to near-total elimination of smoking indoors in key public venues, including restaurants and bars and considerable reductions in smoking in indoor workplaces. The percentage of bars and restaurants where there was observed smoking decreased from near 100% for bars and about 60–71% for restaurants to about 4% for bars and 2–3% for restaurants at the first post-ban measure. Even 5 years after implementation of the ban in hospitality venues, the percentage of observed smoking in restaurants was about 1–2% and smoking in bars was just 6–8%. Similar reductions were evident in self-reported smoking in these venues.

The near total elimination of smoking in these public places where before there was very high or near universal levels of smoking indicate the power of smoke-free laws when they are comprehensive and implemented properly. Previous ITC comparative evaluation of comprehensive vs. partial workplace smoke-free legislation in Ireland, England, and the Netherlands provides evidence that comprehensive smoke-free legislation as implemented in Ireland and England had positive effects on increasing quit attempts and increasing quit success respectively, while partial smoke-free legislation in the Netherlands was not shown to have an impact on quit attempts or quit success [30]. Nagelhout et al. (2011) [17] analysis of the effectiveness of European smoke-free laws confirm the principles for best practice described in the Article 8 Guidelines [9], which call for countries implementing smoke-free laws to engage first in educational programs to inform the public and establishments about the public health harms of second-hand smoke and thus increase public awareness and facilitate support for comprehensive smoke-free laws. The findings here affirm those principles and further, that smoking reductions are sustained over a longer period of time, in this case 4–5 years. In the midst of the overwhelmingly positive effects is a possible concern in one area: among smokers, observed smoking in bars/pubs was slightly higher at the second follow-up than it was at the first follow-up, which points to the need for sustaining and even strengthening efforts at enforcement of the law.

The data on support for smoking bans show that support for the law increases substantially after its implementation, with 77% (74.0–80.2) of smokers supporting the ban in bars, pubs, and cafés and over 87% (85.4–90.0) of smokers supporting the ban in restaurants, slightly less than non-smokers (94% (90.1–96.4)). These findings add to the body of evidence across multiple countries that support for smoking bans in public places increases, especially among smokers, after the implementation of the ban [31]–[33]. It is well established that comprehensive smoke-free laws reduce the perceived social acceptability of smoking [34], [35] and reduce social cues for smoking [36]–[37].

The data also confirm that smoking bans in public places do not lead to displacement of smoking into the home (see Mons et al., 2012, for a review across ITC countries in Europe [18]); indeed they led to an increase in home bans. Mons et al. (2012) analysis of smoking bans in bars across ITC countries in Europe found that positive attitudes towards smoking bans in bars were a significant prospective predictor of having adopted a home smoking ban between pre- and post-legislation survey waves [18].

We also examined support for possible future smoke-free initiatives such as bans on smoking in cars with children in them, which some jurisdictions have implemented in recognition of the very high levels of second-hand smoke produced by smoking in the small volume inside a car [38]–[39]. There was very high support at Waves 2 and 3 among smokers (remained the same from 89% (86.3–90.5) to 89% (86.8–91.1)) and non-smokers (90% (87.1–92.9) to 92% (89.2–94.5)) for banning smoking in cars with children. Laws banning smoking in cars with children have been successfully implemented in a number of jurisdictions, including Cyprus, Bahrain, and Mauritius (which bans smoking in cars with any passenger regardless of age), all provinces in Canada, nearly all of the states and territories in Australia, and three states and many communities in the U.S.

However, there exist lower levels of public support for smoke-free laws governing outdoor terraces, and lack of compliance with the existing law banning smoking in covered or enclosed terraces [40] suggests that additional educational efforts to inform people about the public health rationale for such restrictions would be beneficial. In addition, there will need to be greater attention paid to enforcement in these outdoor, but adjacent areas of hospitality venues.

### Strengths and Limitations

The strength of this study is that the ITC France Survey was conducted on a large nationally representative sample of smokers and non-smokers using a longitudinal design, which allows analyses of changes and to identify factors that are prospectively associated with changes. Such analyses are the focus of future studies.

One possible limitation of this study is in the validity of self-report measures. This is a particular concern when the questions are of a personal nature. However, the survey measures reported in this paper were either not personal (The last time you visited (a restaurant), were people smoking inside the restaurant?) or were those that can only be ascertained through self-report (Do you support or oppose the French total ban on smoking inside restaurants?). Another potential limitation would be in biases due to attrition. However, there were no significant differences in the Wave 1 demographic characteristics (sex, education, income) and key smoking variables (CPD and ever tried to quit) among those who were lost to attrition at Wave 2 and those who completed the Wave 2 Survey. Similarly, there were no significant differences in the Wave 1 responses for noticing smoking, support for bans, and smoking inside bars/restaurants/workplaces among those respondents who participated in Wave 2 and those who did not.

### Conclusions

The results of this longitudinal evaluation study demonstrate the substantial success of France’s comprehensive smoke-free laws, both at one year and five years after the law. Future waves of the ITC France Survey will continue to evaluate support for and compliance with smoke-free laws, as well as long term effects of the law on smoking cessation. The ITC France Survey will also continue its rigorous evaluation of France’s policies in other domains of the FCTC, for example, price/taxation, and health warning labels. Implementing stronger tobacco control policies across the range of the FCTC will be required to combat the recent increase in smoking in France [41], and strong surveillance and evaluation systems are critically important for measuring policy impact and for promoting evidence-based policies [12], [14].
